# A grounded theory study on the influence of sleep on Parkinson’s symptoms

**DOI:** 10.1186/s13104-016-2114-3

**Published:** 2016-06-10

**Authors:** Merel M. van Gilst, Iris C. Cramer, Bastiaan R. Bloem, Sebastiaan Overeem, Marjan J. Faber

**Affiliations:** Department of Neurology, Donders Institute for Brain, Cognition and Behaviour, Radboud University Medical Center, PO Box 9101, 6500 HB Nijmegen, The Netherlands; Sleep Medicine Centre Kempenhaeghe, Heeze, The Netherlands; Eindhoven University of Technology, Eindhoven, The Netherlands; Radboud Institute for Health Sciences, Scientific Institute for Quality of Healthcare, Radboud University Medical Center, Nijmegen, The Netherlands

**Keywords:** Sleep benefit, Parkinson’s disease, Grounded theory, Motor symptoms, Sleep

## Abstract

**Background:**

Upon awaking, many Parkinson’s patients experience an improved mobility, a phenomenon known as ‘sleep benefit’. Despite the potential clinical relevance, no objective correlates of sleep benefit exist. The discrepancy between the patients’ subjective experience of improvement in absence of objective changes is striking, and raises questions about the nature of sleep benefit. We aimed to clarify what patients reporting subjective sleep benefit, actually experience when waking up. Furthermore, we searched for factors associated with subjective sleep benefit.

**Methods:**

Using a standardized topic list, we interviewed 14 Parkinson patients with unambiguous subjective sleep benefit, selected from a larger questionnaire-based cohort. A grounded theory approach was used to analyse the data.

**Results:**

A subset of the participants described a temporary decrease in their Parkinson motor symptoms after sleep. Others did experience beneficial effects which were, however, non-specific for Parkinson’s disease (e.g. feeling ‘rested’). The last group misinterpreted the selection questionnaire and did not meet the definition of sleep benefit for various reasons. There were no general sleep-related factors that influenced the presence of sleep benefit. Factors mentioned to influence functioning at awakening were mostly stress related.

**Conclusions:**

The group of participants convincingly reporting sleep benefit in the selection questionnaire appeared to be very heterogeneous, with only a portion of them describing sleep benefit on motor symptoms. The group of participants actually experiencing motor sleep benefit may be much smaller than reported in the literature so far. Future studies should employ careful inclusion criteria, which could be based on our reported data.

## Background

Parkinson’s disease is a neurodegenerative disorder with motor symptoms primarily characterised by stiffness and slowness of movement. Many patients also experience non-motor symptoms such as cognitive problems, depression and sleep disorders. Although sleep disorders are highly prevalent in Parkinson’s disease [1], there are also patients that report beneficial effects of sleep. Upon awaking in the morning, many patients experience an improved mobility as if they are in a medication-induced “on” state, contrary to what would be expected after a night without medication. This phenomenon is known as sleep benefit [2]. Some Parkinson patients even state to delay or forget their morning dose of medication because of this sleep benefit [3, 4].

In questionnaire studies on sleep benefit, 30–55 % of the patients reports to be familiar with the phenomenon [3–5]. One study described an improvement in clinical motor examination (UPDRS-III) after sleep in patients that experienced sleep benefit [6]. However, results from a recent study by our group on objective motor improvement in sleep benefit showed a different picture. We used two quantitative motor tasks to assess sleep benefit in Parkinson patients. We found no objective sleep-related improvement in Parkinson signs in patients who nonetheless declared to experience subjective sleep benefit [7].

The discrepancy between the patients’ experience of sleep benefit in absence of objective improvement is striking, and raises questions about the nature of the sleep benefit phenomenon [7]. The origin and characteristics of *subjective* sleep benefit are still unclear. Because of the use of closed-end questions in previous sleep benefit research, it is not known what kind of improvement patients refer to when they report sleep benefit. It seems reasonable to make a distinction between (1) a subjective general feeling of improvement after sleep and a (2) specific improvement in actual motor functioning, as probably both are perceived as sleep benefit by Parkinson’s patients [4]. More insight in the subjective experiences of patients who report sleep benefit, could be key in understanding this phenomenon.

Qualitative research methods provide a powerful tool for a more profound understanding of subjective experiences [8–10]. We performed a qualitative interview study to obtain more insight in what patients who report sleep benefit, experience when they wake up. Furthermore, we aimed to identify the factors that may influence the self-reported sleep benefit.

## Methods

### Subjects

**Fig. 1:**
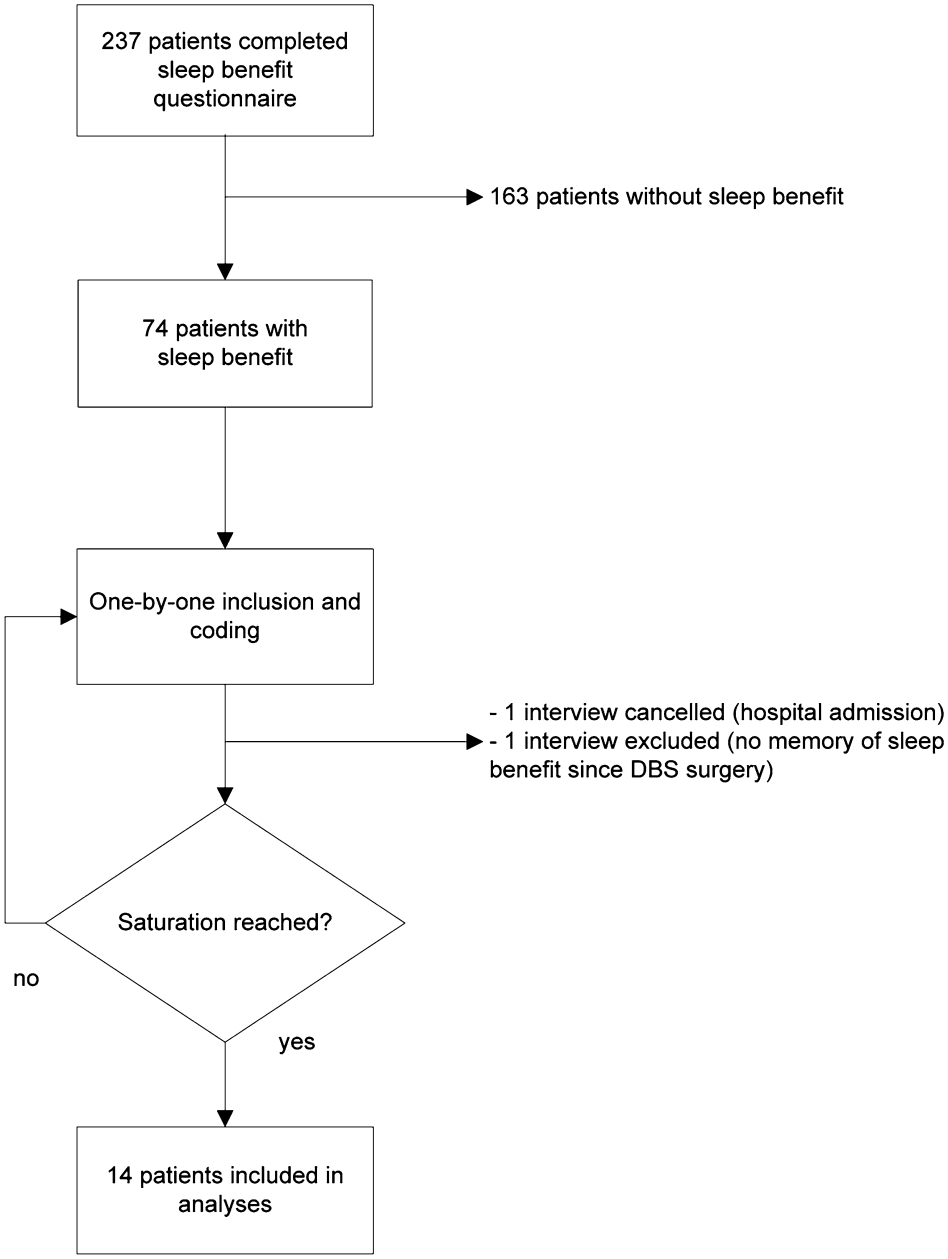
Flow chart patient inclusion

A flow chart of the inclusion process can be found in Fig. 1. Parkinson patients were recruited from a cohort of 237 patients that participated in a previous questionnaire study on sleep benefit [4]. In this questionnaire the following definition/explanation of sleep benefit was used: *sleep benefit isthe experience of a temporary decrease in Parkinson’s symptoms upon awakening after a period of sleep (night or daytime), before drug intake; the patient is feeling as good as “on” (or better)* [11]. For this study we only selected Parkinson patients that reported sleep benefit in the questionnaire (n = 74). We used purposive sampling. With this method, subjects are not randomly selected, but chosen based on individual characteristics. The purpose of this method is to make the sample as diverge as possible. Furthermore, the open-end question in which patients gave a description of their morning experience was leading in the selection: we invited patients who, based on their own description, were most probable—in our clinical view- to experience some sort of sleep benefit. Subjects giving a description indicative of misunderstanding the definition of sleep benefit given in the questionnaire were not invited. Inclusion of subjects and initial coding of the interviews were performed in parallel. New patients were included until data saturation was reached. This means that the last interviews provided no new information and no more new codes emerged from the coding process.

In total, information letters about the study were sent to 16 Parkinson patients. These patients were contacted by telephone to provide general study information and—when interested—to book an appointment for the interview. After telephone consultation, all subjects agreed to participate.

We included patients with idiopathic Parkinson disease, defined according to the UK Brain Bank criteria, Hoehn and Yahr stage I–IV. All patients were fluent in Dutch. Exclusion criteria included current major psychiatric or cognitive disorders. The study was approved by the institutional medical ethical committee (file no. 2014-1355). All participants gave their written informed consent before participating.

### Interviews

For the semi-structured interview with open questions, we used an interview guide. Initially the interview guide only contained a few very wide scoped questions. Participants were invited to tell everything they considered relevant, without steering them into a certain direction.

Participants were asked how they experienced their symptoms at waking up. Both night sleep and daytime naps were evaluated. Questions on day-to-day variation in functioning gave more insight in factors that could influence sleep benefit. Later in the series we added more focussed questions on various specific Parkinson symptoms and aspects of sleep quality. Probing questions were used to follow up whenever necessary. The interviews took place at the participant’s home. Interviews lasted about 30–45 min and were recorded and later transcribed verbatim by the interviewer.

The interviewer (ICC) was trained and experienced in taking interviews. She was new to the field of sleep benefit, as such she was open to whatever the participants presented and had no preconceptions about the phenomenon.

### Analyses

A grounded theory approach was used to analyse the data [8]. The interviews were read and re-read to become familiar with the data. Initial open coding was performed by two researchers (MvG and ICC) independently to increase reliability. All codes were compared and contrasted until consensus was met. This was followed by an axial coding process, in which codes were sorted and classified into recurring themes. All researchers from the multidisciplinary team were involved in the development of the final thematic structure. Generally, differences in interpretation between researchers were small and consensus was rapidly achieved. All ideas and questions that were raised during the analytical process, were registered in memo’s. A computer software package, Atlas.ti (version 7), was used to manage the data.

## Results

A total of 14 Parkinson patients (5 men, 36 %) were included in the analyses. One interview was cancelled because the participant was admitted to the hospital. Another interview was excluded because the participant had surgery for deep brain stimulation (DBS) and did not experience any Parkinson symptoms ever since (Fig. 1). She could not remember much about her symptoms at waking up before the DBS, i.e. the time she completed the inclusion questionnaire.

Age of the participants ranged from 55 to 75 years (mean 61 years). Disease duration (from onset of first symptoms) ranged from 5 to 30 years (mean 13 years). All patients used dopaminergic medication; 13 patients used l-dopa and 8 patients (also) used dopamine agonists.

After coding of the data two central themes emerged. (1) Experiences at waking up, which could be divided in the sub-themes physical functioning and mental functioning and (2) Factors that influenced functioning at awakening, which included sleep related factors and other factors.

### Experiences at waking up

When participants told about their experiences at waking up, these could be divided into two sub-themes; physical functioning and mental functioning, in which participants described their physical/mental abilities at waking up. Both sub-themes had ‘positive’ and ‘negative’ categories, containing codes that indicated functioning that was notably good at awakening or -in contrast- functioning that was affected by the Parkinson’s disease. The physical theme contained codes for different kinds of actions and symptoms, such as getting out of bed, walking and tremor. In the mental theme we identified codes for different aspects of mental functioning, such as feeling relaxed, feeling rested and feeling energetic.

Based on the co-occurrence of different codes within this theme, we distinguished three interpretations of sleep benefit: (1) patients that described motor sleep benefit (n = 6), (2) patients without sleep benefit (n = 5) and (3) patients that experienced unclassified sleep effects, i.e. some sort of improvement that was not clearly sleep benefit (n = 3).

#### Motor sleep benefit

The six patients in this group clearly described a decrease in Parkinson-specific motor symptoms. At waking up they find it easier to perform all sorts of movements, such as getting out of bed, walking, getting dressed, writing and household tasks. Furthermore, some patients experienced less tremor and freezing of gait.

*“I just get out of bed and walk. I don’t have to sit on the side of the bed first, I can get up without effort.” (P5, night)*

*“Yes, then my handwriting is fine, however that expires within half an hour, then it’s over.” (P6, night)*

*“the first half hour in the morning, I would describe it as feeling ‘on’” (P6, night)*

*“But the tremor, I don’t feel, I don’t have it when I wake up” (P14, night)*

*“Well, I go to the kitchen for example, and it is easy to clean the countertop, or eh, to make sandwiches, that kind of things. And I do not stick to the ground.” (P1, nap)*

Although moving was easier, most of these patients *did* experience stiffness when getting out of bed.

*“Getting dressed is easier, but walking is stiff. Tasks in the kitchen are trouble*-*free” (P1, night)*

*“I feel a little stiff, but that’s it. In the morning I’m at my best” (P4, night)*

Patients with motor sleep benefit described a clear limited duration of the effects, ranging from 30 min to 2 h. All patients experienced an improvement in motor functioning after night sleep. Some patients also reported motor benefit of an afternoon nap or when waking up during the night. Many patients also reported a mental benefit of sleep, mostly in terms of ‘feeling well rested’.

#### No sleep benefit

These 5 patients did not meet the definition of sleep benefit for various reasons. For example, some patients experienced many symptoms at awakening. Therefore, they took their morning medication in bed and waited in bed until it started working. The described ease with getting out of bed was clearly a medication effect rather than a sleep effect. Other patients needed their medication at waking up, however, overall they felt better in the morning than in the afternoon. There was also a patient who described that a good night of sleep was very beneficial for functioning during the whole next day. So the effect was not specific for the first moments after waking up, but rather an all day ‘bonus’ that worked on top of the normal medication.

#### Unclassified sleep effects

There were three patients that did not fit in either the motor sleep benefit or the no sleep benefit group. These patients reported to feel good at waking up. However this improvement mostly seemed to be a mental rather than a physical change. Patients described feeling relaxed, energetic, clear headed or peaceful. This state of mind was reflected in overall functioning, but Parkinson specific motor symptoms were present explicitly at awakening.

*“That is not really a physical thing, but in my head, I feel really relaxed. Whatever happens, I just let it happen without fighting it. So there is more comfort in my body maybe. I don’t worry so much or think ‘whatever’. And then I am able to get up calmly.” (P2, night)*

*“Yes, yes, my body is relaxed, it feels very pleasant, a positive feeling. It has something to do with mood. That’s the biggest difference, when you feel relaxed, your body, your mood is ten times better. That is the largest benefit.” (P2, night)*

*“It is like calmness comes into your body, relaxation in your head. And I am a busy bee, but then it is just, I can let everything go. Like recharging, I feel invigorated.” (P8, nap)*

*“See, before I take a nap, I’m exhausted, especially in my head. I close my eyes and fall asleep immediately. Well, if I’m tired, my whole body, everything is difficult. But when I wake up I feel as fit as a fiddle so to say (laughs), then it’s just fine.” (P9, nap)*

In this group, the described beneficial effects of sleep could occur after night sleep, an afternoon nap or both. The benefit started at the moment of waking up, however, these patients did not indicate a clear duration of the phenomenon.

One patient took her medication in bed and slept until the medication started working. Sometimes she woke up feeling exceptionally good. Other mornings getting out of bed was very troublesome.

*“Well, when I wake up feeling relaxed, taking a shower is easier. I can just move more easily. […] And when I don’t wake up feeling relaxed, when I start stiffening, that uncomfortable feeling, then washing myself is difficult.” (P9, night)*

### Factors that influenced functioning at awakening

#### Influence of sleep factors

There was large variation in self described sleep quality between patients. We could not identify any concurrent patterns in the different sleep benefit groups. Notably almost all patients described to experience REM sleep behaviour-like symptoms, such as vivid dreaming, (violent) movements and speech during sleep.

Some patients reported factors that could influence the magnitude of the beneficial effects of sleep. Total sleep duration, number of awakenings, presence of vivid dreams and bedtime were mentioned. However, there were also patients who denied the influence of these factors or were not aware of any contributing factors at all.

Some patients experienced the same amount of motor benefit after an afternoon nap as after a night of sleep. Others reported to benefit from a nap as well, but to a lesser extent. Some patients mentioned that this could be because naps have a shorter duration. Only a number of patients experiencing unclassified sleep effects had more benefit of a nap than of a night of sleep.

#### Other factors

Many patients, both with and without sleep benefit, described a negative influence of stress on their symptoms at waking up. If they had an appointment in the morning and had to hurry through their morning rituals they felt less comfortable and experienced more Parkinson’s symptoms.

On the other hand, there was also one patient that praised the silence of the night as the best influence on his symptoms:

*“Yes, the difference is that there is nothing around me. It’s dark, there is nobody there, nobody active and I can just without any, anything around me, there is no disturbance. […] no influence from outside, that’s easier.” (P2, night)*

Some patients described that not only sleep or a nap had positive effects, but also taking rest, without sleeping could reduce tremor or give more energy.

## Discussion

In this grounded theory study, we interviewed patients with self-reported sleep benefit about their functioning at waking up and about the factors that could influence their abilities at awakening. We showed that some, but certainly not all, patients with self-reported sleep benefit experience a temporal decrease in their Parkinson motor symptoms. Several patients did not meet the definition of sleep benefit at all. Others experienced beneficial effects of sleep, however, not a specific decrease of motor symptoms. There were no general sleep related factors that influenced the presence of sleep benefit. Other factors that were mentioned to influence functioning at awakening were mostly stress related.

For this study we selected patients that, by all available means, were likely to experience sleep benefit, selected from a large cohort of patients that reported sleep benefit in a questionnaire. Surprisingly, a substantial part of the interviewed patients did not meet the definition of sleep benefit, that was presented in the questionnaire, for various reasons. In all these cases, patients did not take into account a crucial part of the definition of sleep benefit. They mainly disregarded the statement that sleep benefit is experienced *before* the intake of medication or that sleep benefit is a *temporal* effect, specific for the moment of waking up.

In previous studies on objective motor improvement in patients with sleep benefit, questionnaires were used for the selection of participants [6, 7]. Our data show that this inclusion method may result in a very heterogeneous group of participants. Probably patients without sleep benefit or with unclassified sleep effects participated in those studies as well. This may –in part- explain why no motor effects of sleep benefit have been found. It would be interesting to study whether patients with self-described motor sleep benefit show objective motor improvement after sleep. Our data show that the careful inclusion of patients in such a study is very important. Based on these interviews we could not find differentiating factors for motor sleep benefit. In future studies an extensive interview will be the best way to distinguish patients with sleep benefit from those without.

Three participants did not describe classical motor sleep benefit. However, they did experience some beneficial effects of sleep, mostly mental benefit. The mental improvement described by these patients was not a Parkinson specific effect of sleep, but rather a subjective general feeling of improvement. It could be argued whether this should be regarded a form of sleep benefit. For these patients, it directly benefited their coping with the disease. Therefore, we think that also this phenomenon could be clinically interesting, but the underlying mechanisms are probably different from motor sleep benefit. So, it is important to make a clear distinction between both phenomena.

We could not find sleep related factors that clearly influenced the occurrence of sleep benefit. Some participants did experience a relation between sleep quality and the amount of sleep benefit, however, there were large individual differences. Based on the small number of patients with motor sleep benefit in this sample, we cannot draw conclusions on this matter.

The other factors that participants described that influenced their functioning at waking up were not specific for sleep benefit. Factors such as stress were also considered disabling during the rest of the day and are often reported by Parkinson’s patients [12]. On the other hand, some participants described feeling relaxed, peaceful or clearheaded after sleep, as if sleep had a stress reducing effect. In fybromialgia and rheumatoid arthritis, good sleep has a beneficial effect on coping with stressful events [13]. So, maybe in these cases sleep does not have a direct effect on functioning, but the effect is mediated by a reduction of stress or negative affect. Some participants also described beneficial effects of taking rest, without sleeping. This could have a stress and fatigue reducing effect as well.

In studies on objective motor function in sleep benefit, patients had to perform motor tasks directly at waking up [6, 7]. This could have been consciously or unconsciously stressful for the participants. At least the experimental setting differed from their standard morning routine, which many patients considered important in this study. This could have contributed to the lack of motor effects found in the quantitative studies on sleep benefit.

Notably, many patients, also amongst those with motor sleep benefit, described feeling stiff at waking up. However, as some patients added, this could also be a general sign of aging instead of a specific Parkinson symptom. Some participants noted the same stiffness at waking up with their spouses, who were not diagnosed with Parkinson’s disease.

For some participants it was difficult to find the exact words to describe their functioning at awakening. To help these participants, the interviewer asked probing questions on morning routines and activities. Especially on highly subjective topics, such as mental benefit, interpretation of the statements was not always unequivocal. Nevertheless, these interviews provided very divergent and exemplifying stories that could have not been retrieved using standardized questionnaires.

In conclusion, we showed that not all Parkinson’s patients with self-reported sleep benefit do experience a specific reduction in motor symptoms after sleep. The group of patients convincingly reporting sleep benefit in a questionnaire turned out to be very heterogeneous, with only a portion of the patients describing motor sleep benefit. Although the results of qualitative research cannot be generalized to the whole Parkinson’s population, it seems probable that the group of patients actually experiencing motor sleep benefit is far smaller than thought so far, based on previous questionnaire studies.
